# Supplementary material for: Quality of life, self-reported outcomes and impact of education among people with moderate and severe hemophilia A: An integrated perspective from a Latin American country
Source: PLoS One. 2023 Jul 6;18(7):e0287972. doi: 10.1371/journal.pone.0287972 (PMC10325071; doi:10.1371/journal.pone.0287972)
Supplement: S1 File — (PDF) [file pone.0287972.s001.pdf]

Test de conocimientos para pacientes en condición de Hemofilia
Campamento Nacional de Hemofilia 2019

Lee con atención las siguientes preguntas y selecciona con una x la respuesta que consideres correcta:

1. La hemofilia es una enfermedad que se caracteriza por:
   A. La falta de capacidad para coagular la sangre
   B. Un desorden que se caracteriza por excesiva coagulación
   C. Una enfermedad en la que hay una producción exagerada de células de la sangre

2. ¿Por qué las personas con hemofilia a veces sangran durante más tiempo?
   A. Por exceso de unas células llamadas plaquetas
   B. Por falta de unas células llamadas glóbulos rojos
   C. Por falta de un factor de coagulación o un nivel bajo

3. Verdadero o falso:
   A. Las personas que tienen bajos niveles de factor VIII (8) tienen hemofilia B
   B. Las personas que tienen bajos niveles de factor IX (9) tienen hemofilia A
   C. La hemofilia puede ser leve, moderada o severa, dependiendo del nivel de factor de coagulación

4. ¿Cómo les da hemofilia a las personas?
   A. Las personas pueden contagiarse de hemofilia
   B. La hemofilia se transmite a través de los genes de los padres
   C. El gen de la hemofilia lo lleva el cromosoma Y

5. Verdadero o falso:
   A. Uno de cada tres bebés no tiene historia familiar de hemofilia
   B. Una persona nacida con hemofilia la tendrá durante toda la vida

6. Completar usando las siguientes palabras: hemorragias, músculos, cirugía, articulaciones, espontáneas:
   A. Las __________ en la hemofilia pueden ser visibles o no. Pueden ocurrir después de un trauma o una _________. También pueden ocurrir sin motivo aparente y se llaman _________. A medida que los niños crecen, las hemorragias espontáneas son más comunes; éstas afectan las _________ y los _________.

7. Con respecto a las hemorragias articulares:
   A. Siempre se presentan después de un trauma
   B. Empieza como hormigueo y calor
   C. Se presentan con mayor frecuencia en las manos

FUNDACIÓN BIOS
COMITE DE ETICA
29 NOV. 2019

8. De las hemorragias graves o que ponen en peligro la vida:
   A. La sangre en la orina es común en casos de hemofilia severa, aunque pocas veces es peligrosa
   B. Las hemorragias en la cabeza no constituyen una causa importante de muerte
   C. Las hemorragias en la garganta no son relevantes

9. ¿Qué son los inhibidores?
   A. Los inhibidores son anticuerpos (proteínas) que el cuerpo desarrolla para combatir cosas que percibe como "ajenas"
   B. Los inhibidores no afectan al tratamiento
   C. Se presentan sobretodo en pacientes con hemofilia leve

10. ¿Qué puede hacerse para mantenerse saludable?
    A. Ejercítese y manténgase en forma
    B. El ejercicio es perjudicial y agrava las lesiones en las articulaciones
    C. La salud oral no contribuye al éxito del tratamiento

11. ¿Cómo se tratan las hemorragias con terapia de reemplazo de factor?
    A. El factor de coagulación puede suministrarse por la boca
    B. No es necesario hacer nada
    C. Puede tratarse inyectando el factor de coagulación faltante en una vena

12. Frente a una hemorragia en una articulación, se debe:
    A. Hielo, elevación, reposo, compresión
    B. Calor, trotar, sobarse, flexionar
    C. Apoyar, aplicar cremas, rezar, salir corriendo

13. En caso de tener un sangrado:
    A. Se acuesta, descansa y espera un rato que el sangrado mejore por sí mismo.
    B. Usa la dosis de profilaxis que tiene en la nevera para contener el sangrado.
    C. Llama o se dirige al centro de atención de urgencias.

14. ¿Qué es el autocuidado para ti?:
    A. El cuidado que me brinda mi madre y parientes cercanos.
    B. El cuidado y las indicaciones que recibo de mi equipo médico.
    C. Las acciones y prácticas que realizo de manera responsable para cuidar de mí.

15. ¿Por qué crees que es importante organizarse como comunidad de hemofilia?
    A. Porque nos encontramos y pasamos bien juntos.
    B. Porque conocemos mejor las fortalezas y necesidades de las personas con hemofilia y sus familias.

FUNDACIÓN BIOS
COMITE DE ETICA
29 NOV. 2019

C. Porque es necesario trabajar juntos por mejorar el tratamiento y las condiciones de vida de las personas con hemofilia y sus familias.
D. Todas las anteriores.

FUNDACIÓN
BIOS
COMITE DE ETICA
29 NOV. 2019
