# Supplementary material for: Quality of life, self-reported outcomes and impact of education among people with moderate and severe hemophilia A: An integrated perspective from a Latin American country
Source: PLoS One. 2023 Jul 6;18(7):e0287972. doi: 10.1371/journal.pone.0287972 (PMC10325071; doi:10.1371/journal.pone.0287972)
Supplement: Attachment — Submitted filename: PONE-D-22-24662_Response to Reviewers.docx [file pone.0287972.s003.docx]

**Response to Reviewers**

**Reviewer #1 Comments:**

*We are grateful for the expert comments and excellent advice we have received. In responding your respective comments, we used abbreviation “A” in place of “authors”. The point-by-point responses to the comments are as follows:*

1. The English is very good, but there are occasions where the sentence construction isn't quite right. There are also a small but frequent number of text errors which should be addressed. It would benefit the manuscript for you to have it reviewed by a language/copy editing service if possible.

*A/ The manuscript was reviewed by an editing service (American Journal Experts) according to the reviewer suggestions.*

2. You have mentioned the relatively small number of participants who were able to self infuse but have not discussed this further. It would be interesting and would benefit the paper if you were to discuss this issue further, including why there are so few. what are the barriers, and how self infusion rates might be improved. It would also be interesting to see if there were any differences between the QoL of those who were able to self infuse, those who were on Prophylaxis administered by others and those who were on demand etc.

*A/ The Discussion was reviewed to address the low self-infusion rates observed in the study in the context of the Colombian health system and how education is key to increase the rates among PwHA in our country, with the inclusion of additional references.*

*Regarding the differences between the QoL among those who self infuse, this information is not available since it was captured in the qualitative portion of the study through the focus group and due to data privacy reasons, we’re not able to match the EQ5D responses with the individual responders from the focus group. Furthermore, the PROBE questionnaire does not specifically ask about self-infusion (only up to the primary treatment regime and where do the patients receive such treatment). Therefore, we are not able to perform this analysis with the quantitative information that is available. We have clarified this in the Discussion as a study limitation*
